# Emerging Opportunities for 2D Materials in Neuromorphic Computing

**DOI:** 10.3390/nano13192720

**Published:** 2023-10-07

**Authors:** Chenyin Feng, Wenwei Wu, Huidi Liu, Junke Wang, Houzhao Wan, Guokun Ma, Hao Wang

**Affiliations:** 1 Hubei Yangtze Memory Laboratories, Wuhan 430070, China; 2 Institute of Microelectronics and Integrated Circuits, School of Microelectronics, Hubei University, Wuhan 430062, China

**Keywords:** neuromorphic computing, 2D materials, memristor, memtransistor

## Abstract

Recently, two-dimensional (2D) materials and their heterostructures have been recognized as the foundation for future brain-like neuromorphic computing devices. Two-dimensional materials possess unique characteristics such as near-atomic thickness, dangling-bond-free surfaces, and excellent mechanical properties. These features, which traditional electronic materials cannot achieve, hold great promise for high-performance neuromorphic computing devices with the advantages of high energy efficiency and integration density. This article provides a comprehensive overview of various 2D materials, including graphene, transition metal dichalcogenides (TMDs), hexagonal boron nitride (h-BN), and black phosphorus (BP), for neuromorphic computing applications. The potential of these materials in neuromorphic computing is discussed from the perspectives of material properties, growth methods, and device operation principles.

## 1. Introduction

As Moore’s law approaches its limit, computing technology is facing an urgent demand for new computational methodologies to overcome challenges in power and computational efficiency, particularly in artificial intelligence and machine learning. The human brain contains 10^14^–10^15^ synapses, which is more efficient and powerful than any current digital computers [1,2,3]. It presents itself as an ideal prototype for novel computing architectures [4,5]. Therefore, neuromorphic computing has gained a lot of attention recently.

Neuromorphic computing aims to mimic the way the brain processes information by using electronic circuits or software architectures that emulate the behavior of neurons and synapses [6,7,8]. The ultimate goal is to engineer electronic systems that obtain the computational efficiency, learning capabilities, and fault tolerance of biological brains, all within a compact and efficient design [9]. Over time, researchers have been inspired by biological neurons, leading to the development of multiple generations of neural network architectures [10]. For instance, in 1943, McCulloch and Pitts combined neuron models to propose what is now known as the McCulloch–Pitts (MP) model, a precursor to modern neural network structures [11]. Notably, in 1949, the introduction of the Hebbian theory established the concept of variable synaptic weights and synaptic plasticity, laying a foundational understanding to enable learning in neural networks [12]. The first generation of these networks was the perceptron, a single-layer structure encompassing only input and output layers. Its activation function was simple, restricting it to basic linear classifications. Based on this, artificial neural networks (ANNs) were developed [13]. These included an added hidden layer between the input and output, alongside a more complex, nonlinear activation function, enabling nonlinear classification tasks. The most iconic of this generation is the feed-forward neural network (FFNN), which transmits information unidirectionally, from the input, through the hidden layer(s), and, finally, to the output [14].

Building on this foundation, researchers have proposed more advanced architectures like deep neural networks (DNNs) [15,16], which boast a multitude of hidden layers, and recurrent neural networks (RNNs) which incorporate feedback connections to handle temporal data [17,18]. While DNNs harness their profound learning potential from their extensive structures, RNNs are adept at managing sequential data and dynamic information. Furthermore, to expedite an RNN’s training times, reservoir computing (RC) was introduced [19,20,21]. RC offers a streamlined training approach, where the central reservoir matrix is randomly initialized and remains static post-creation. Consequently, only the output layer undergoes training, making RC notably faster than conventional RNN methods.

While ANNs and their advanced versions exhibit robust self-learning capabilities, they are constrained by their foundation of traditional silicon CMOS chips governed by the von Neumann architecture [22,23]. This architecture consumes significantly more power compared with the human brain and does not emulate the intricate operations of neurons and synapses [24]. To bridge this gap, researchers proposed third-generation neural networks: spiking neural networks (SNNs) [21]. Unlike their predecessors, SNNs transmit and process information using current or voltage pulses, closely mirroring the operations of biological neurons. These pulses, containing temporal information, equip SNNs to efficiently handle time-series data and dynamic information. However, due to the asynchronous nature of SNNs, they are not optimal for traditional silicon-based CMOS chips, highlighting the need for innovative neuromorphic devices.

Artificial neuromorphic devices stand apart from traditional silicon-based CMOS devices, which are limited to two switching states and require sequential calculations. Additionally, in traditional devices, data storage and processing areas are segregated. Neuromorphic devices, inspired by brain neurons, boast the following attributes: 1. variable bionic synapse weights, mimicking synaptic plasticity in the brain; 2. low power consumption, achieving efficient information processing; 3. massive parallelism: allowing concurrent data processing; and 4. unified storage and operations: combining data storage and computational tasks at the same site. Several emerging memory technologies show promise in neuromorphic computing, such as resistive-change memory, phase-change memory, magnetoresistive memory, floating-gate field-effect transistors, and three-terminal ferroelectric transistors. In these devices, one terminal imitates the presynaptic input (axon), and another mirrors the postsynaptic output (dendrite). By applying voltage pulses to these terminals, the channel conductance (or synaptic weight) can be modulated, simulating the functionalities of a biological synapse.

Synaptic connections are the fundamental units that link all neurons in the brain, serving as bridges between neurons [21,25]. They transmit neural signals via action potentials (intracellular) and neurotransmitter/electrical signals (extracellular), adjusting synaptic weights. These changes in synaptic weights are also known as neuronal plasticity, which is further divided into short-term potentiation (STP), short-term depression (STD), long-term potentiation (LTP), long-term depression (LTD), spike-timing-dependent plasticity (STDP), and spike-rate-dependent plasticity (SRDP) [26,27,28,29]. Short-term plasticity refers to the adaptability of synapses over a brief period. After a synaptic neuron releases neurotransmitters, the efficacy of synaptic transmission may temporarily increase (STP) or decrease (STD) [30]. These short-term changes in plasticity can affect the intensity and timing characteristics of neural signal transmission. LTP refers to the enhancement effect on synapses after repeated stimulation, increasing the efficiency of synaptic connections. When the synapses between neurons are repeatedly activated, the synaptic transmission efficacy gradually strengthens, and this enhancement can last for an extended period. On the other hand, LTD refers to the inhibitory effect after repeated stimulation, weakening the efficiency of synaptic connections. Contrary to LTP, LTD leads to a gradual decline in synaptic transmission efficiency. Like STDP, it is a type of Hebbian learning mechanism with temporal asymmetry. It is commonly thought to be the basis for learning and information retention in the brain. The resistive switching in memristive devices is triggered by external stimuli, often involving physical or chemical processes like carrier generation/recombination and redox reactions. The combined effects of these mechanisms provide the brain’s neural signal transmission with a high degree of plasticity and adaptability [1,30]. By regulating synaptic plasticity and the strength of signal transmission, the brain can achieve information storage, processing, and learning, which are crucial for human cognition and the learning process.

To build neuromorphic devices capable of parallel processing large data volumes with remarkable plasticity and efficiency, researchers, globally, are exploring suitable materials. Two-dimensional materials, in particular, have garnered interest due to their distinctive properties, offering new avenues for enhancing the precision and integration of neuromorphic devices.

## 2. Overview of 2D Materials

**Figure 1:**
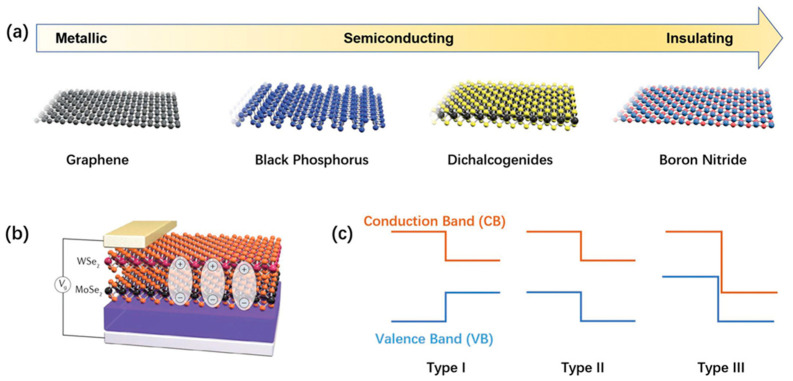
(**a**) Different types of representative 2D materials. (**b**) Heterostructures prepared from 2D materials. (**c**) Energy band diagrams of three heterostructures. Reproduced with permission from [34]. Copyright (2016) by Springer Nature.

Since the successful preparation of single-layer graphene in 2004, two-dimensional (2D) materials have garnered attention worldwide [31]. Over nearly two decades, researchers have extensively studied 2D materials such as graphene, graphene oxide, hexagonal boron nitride (h-BN), transition metal disulfides (TMDs), black phosphorus (BP), and overmetallic oxides (TMOs) [32,33]. These materials span various categories, including insulators, semiconductors, semimetals, metals, and superconductors, thereby offering numerous options for device designs (Figure 1) [34,35]. Recent research has explored the potential of 2D materials for neuromorphic computing due to their distinct properties. This review concentrates on the application of layered 2D materials in brain-like computing. In this section, we will discuss the benefits of 2D materials and their heterostructures, as well as their synthesis and fabrication techniques.

### 2.1. Unique Properties of 2D Materials

**Figure 2:**
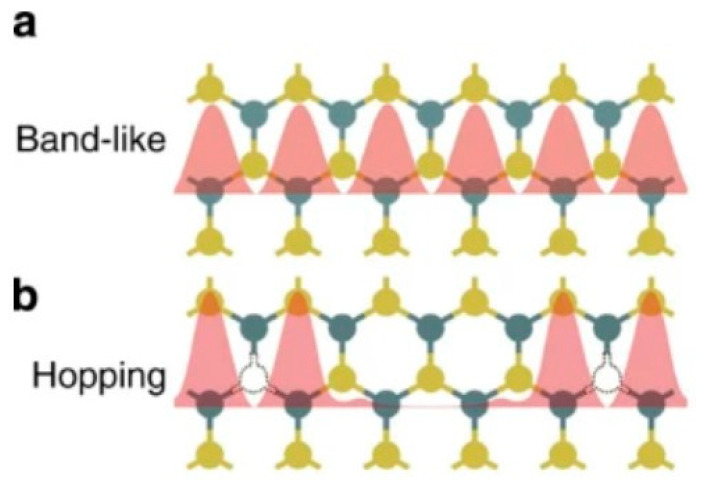
Schematic representation of the electron transport mechanism in perfect and defective MoS_2_. In perfect MoS_2_ (**a**), the electron density is periodically distributed in space, and the transport is banded. However, in defective MoS_2_ (**b**), the electrons are transported in a hopping manner while clustered in the defect attachment. Reprinted (adapted) with permission from [43]. Copyright (2015) by the American Chemical Society.

Two-dimensional materials, or van der Waals materials, are characterized by strong covalent bonds within their layers and weak van der Waals forces between these layers [36]. By using a taped substrate, these materials can be peeled down to a single atomic layer without losing their structural integrity [37]. Such materials effectively mitigate the short-channel effect and can achieve impressive carrier mobility. For instance, graphene encapsulated with h-BN can reach mobilities of 120,000 cm^2^/v·s, while a 10 nm thick black phosphorus film can achieve 1000 cm^2^/v·s [38,39]. These properties facilitate high switching speeds, substantial switching ratios, reduced operating voltages, and low power consumption in 2D-material-based devices [40,41]. Using techniques like mechanical transfer or chemical vapor deposition, 2D materials can be applied to various substrates, making them compatible with conventional CMOS technologies. Additionally, their physical properties can be tailored using methods such as defect engineering, electrostatic doping, chemical embedding, and stress–strain [42]. Among these, structural defects like point defects and grain boundaries play a vital role in tuning intrinsic material properties. Molybdenum disulfide (MoS_2_), for instance, often undergoes structural defect modulation [43]. Several methods, including growth environment adjustments and post-growth electron beam/plasma excitation, allow modulation [43]. Such techniques can influence MoS_2_’s electrical, electrochemical, and optical properties. As depicted in Figure 2, sulfur vacancies in MoS_2_ act as electron donors, introducing localized donor states in the bandgap [43,44,45]. In neuromorphic computing, these vacancies can serve as ion migration channels. When MoS_2_ is heated in atmospheric air, it integrates with oxygen to form a MoS_2−x_O_x_ film. Due to the thermomigration effect, oxygen ions either fill sulfur vacancies or move away, modulating the material’s resistive states. Figure 2 illustrates the electron transport mechanisms in both defect-free and defective MoS_2_. In the former, the electron density is spatially distributed, and the transport is banded. In contrast, defective MoS_2_ exhibits electrons hopping between clustered defects. Generally, the formation or annihilation of conductive channels in 2D materials stems from oxidation, reduction, or metal atom migration, often linked to material defects [46,47]. Such migration typically occurs perpendicular to the material plane, necessitating structural defects [46,48]. By regulating these defects, one can control the conductive channels and the material’s resistive state.

Under a bias voltage, 2D materials exhibit the formation and dissolution of conductive channels. This dynamic is driven by oxidation, reduction, or metal atom migration within these materials. The emergence or disappearance of these channels is tightly linked to defects in the 2D materials [46]. Typically, migration is perpendicular to a material’s plane and is dependent on the structural defects. By adjusting these defects, one can modulate these conductive pathways and consequently control the material’s resistive state.

**Figure 3:**
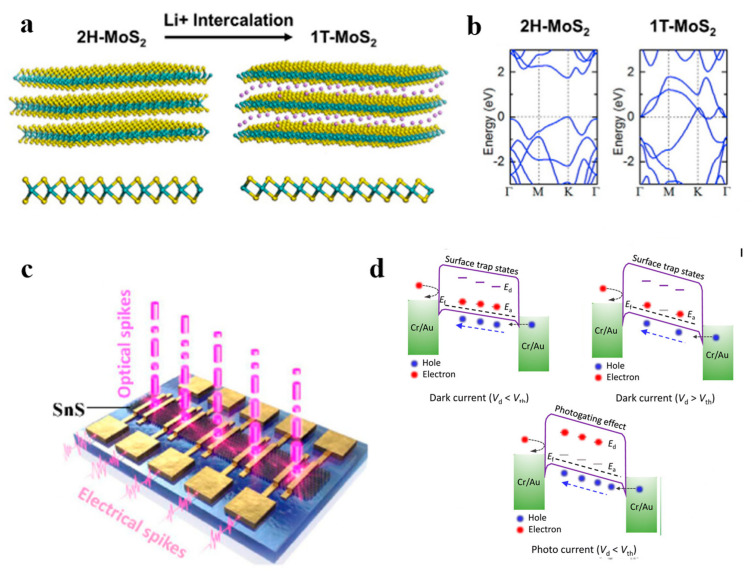
(**a**) 2H-MoS_2_ forms 1T-MoS_2_ via phase transition generated by Li+ ion embedding. Reprinted (adapted) with permission from [49]. Copyright (2016) by the American Chemical Society. (**b**) Energy band diagrams of 2H-MoS_2_ (semiconductor) and 1T-MoS_2_ (metal). (**c**) Schematic diagram of the structure of the SnS-based memristor. (**d**) Schematic diagram of the operating principle of the SnS-based memristor. Reprinted with permission from [46]. Copyright (2021) by the American Association for the Advancement of Science.

Scientists have deeply explored the phenomenon of phase transition in two-dimensional materials. Such materials can toggle between distinct crystalline phases when triggered by specific stimuli. Devices based on phase-transition materials can thus shift from a high-resistance state (HRS) to a low-resistance state (LRS). Due to their minuscule thickness at the atomic level, 2D materials are especially responsive to external influences, making them prime candidates for phase transitions. Given their unique properties, 2D materials can emulate an array of synaptic activities. Notably, techniques have been developed to induce phase transitions in 2D materials, especially in 2D transition metal sulfides (TMDs). As illustrated in Figure 3a,b, introducing lithium ions transitions the original 2H-MoS_2_ into the 1T-MoS_2_ form, marking a shift from semiconductor to metal [49,50]. This transition might be attributed to electrons from lithium ions being attracted to the 4d orbitals of 2H-MoS_2_, leading the 2H phase to destabilize and convert into the 1T phase.

In recent research, the charge trapping effect in two-dimensional van der Waals heterostructures has gained attention for its potential to modify device conductivity. This strategy employs a charge-trapping layer, termed the weight control layer (WCL), to facilitate electron trapping or release [51]. Within 2D van der Waals heterostructure devices, carrier generation, trapping, and release under external stimuli can simulate neuron functions, such as STP/STD and LTP/LTD. Electron trapping or release in such synaptic devices can alter the breadth of the tunneling barriers. Kumar et al. highlighted the creation of potent dual-ended memories using layers of zinc oxide (ZnO) and WS_2_, crafted via an RF sputtering technique [52]. The space between the ZnO and WS_2_ layers creates a conductive porous medium, leading to ZnO defects, which then act as a unique charge-trapping/untrapping layer. Similarly, Vu et al. detailed a memory device featuring vertically aligned van der Waals heterostructures of MoS_2_/h-BN/graphene [53]. This device, built on a charge-trapping/untrapping mechanism, uses graphene as a WCL. Electron microscopy and X-ray spectroscopy revealed the device’s intricate layers. The heterostructure-based device they introduced capitalizes on the charge storage capacity of 2D MoS in graphene, which interacts with the tunneling likelihood through svelte graphene. The device showcases a broad dynamic range, prolonged data retention, reliable endurance, varied conductivity levels, and an impressively low off-current, leading to a remarkable switching ratio. Lastly, Sun et al. unveiled a computational device resembling brain functions, founded on two-dimensional p-type SnS [46]. As depicted in Figure 3c,d, the 2D SnS contains Sn and S vacancies, positioned within the SnS bandgap as acceptor and donor states. When the applied voltage is below the threshold, electrons in the SnS predominantly latch onto Sn vacancies. However, when the voltage surpasses this threshold, the ensnared electrons combine with the vacancies, curtailing the current in this p-type semiconductor device. This mimics the fleeting plasticity observed in synapses.

### 2.2. Synthesis Methods for Two-Dimensional Materials

Various techniques exist for the synthesis of two-dimensional (2D) materials, broadly categorized into top-down and bottom-up approaches [54]. Top-down techniques synthesize 2D materials by extracting a single or a few atomic layers from a larger bulk, typically using external forces like mechanical strain or ultrasound. In contrast, bottom-up approaches involve creating 2D structures by layering atoms onto a base via thermochemical or other chemical processes. Common methods include mechanical exfoliation, chemical exfoliation, chemical reactions, and thermochemical vapor deposition (CVD). Additionally, techniques like laser exfoliation, carbon heat reduction, and high-energy electron beam exposure have been suggested for crafting 2D materials [55]. 

The technique of mechanical exfoliation, pivotal in the production of graphene, was pioneered by Novoselov and Geim in 2004 as a means to create 2D materials [37]. Owing to its straightforwardness and cost-efficiency, mechanical exfoliation has become a favored method for producing 2D materials. This technique preserves the crystal structure and inherent properties of a material by allowing the extraction of one or multiple layers directly. For graphene production, a sheet is peeled from highly oriented pyrolytic graphite (HOPG) using ordinary transparent tape, and this separated layer is subsequently moved to a chosen substrate. Following its success with graphene, micromechanical exfoliation has been employed to produce non-carbon 2D materials, notably those similar to TMDs. Numerous studies detail the synthesis of materials like MoS_2_, MoSe_2_, WS_2_, NbSe_2_, and WSe_2_ using this method. Moreover, successful mechanical extraction of BN has also been documented. Notably, 2D MoS_2_ has been spotlighted as a promising candidate to supersede silicon in future CMOS technologies [56,57]. Mechanical exfoliation is both cost-effective and advantageous for foundational studies, yet producing 2D materials on a grand scale remains a significant challenge.

**Figure 4:**
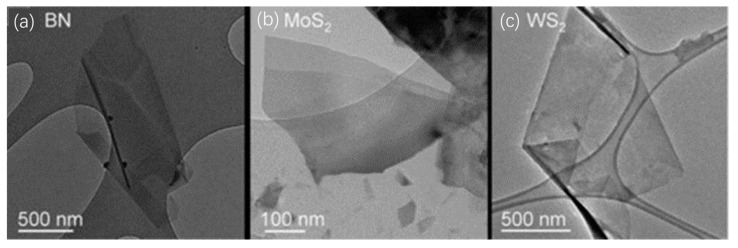
TEM images of BN (**a**), MoS_2_ (**b**), and WS_2_ (**c**). Reprinted with permission from [58]. Copyright (2021) by the American Association for the Advancement of Science.

Another prominent approach to obtaining 2D materials via top-down techniques is the chemical/solvent exfoliation process. In this method, bulk materials are transformed into 2D nanosheets via ultrasonic processing, followed by dispersion in certain liquid solvents for centrifugal separation [58,59]. The application of low-intensity ultrasonic waves facilitates the peeling off of the materials into ultra-thin 2D nanosheets. The number of atomic layers, ranging from 5 to 20, in the resultant 2D materials is contingent upon the inherent structure of the starting material and the choice of organic solvent during centrifugal ultrasonication. Moreover, the finesse of these 2D materials is influenced by both the duration of the ultrasonic exposure and the centrifugation speed. Illustrative TEM images of h-BN, MoS_2_, and WS_2_, obtained using this method, can be seen in Figure 4a, Figure 4b, and Figure 4c, respectively [58]. While the chemical/solvent exfoliation technique is esteemed for its adaptability and potential for large-scale production, there are challenges to surmount. Achieving a consistent dispersion of the material in the solvent is complex, given that the evenness relies on the solvent’s unique surface tension properties. Additionally, the presence of an ionic contaminant layer, resulting from impurity ions physically adhered to the 2D sheets, can impair both the electrical and thermal characteristics of the final material.

Chemical synthesis serves as a bottom-up technique for crafting 2D materials. Atoms or molecules are methodically deposited onto a substrate, leading to the formation of a 2D configuration. As the chemical/solvent stripping approach made strides in producing 2D materials, the chemical synthesis technique soon garnered significant research interest [60]. In essence, this aqueous chemical procedure facilitates large-scale, cost-effective manufacturing of 2D TMDs. Notably, Sekar’s team introduced a procedure for developing 2D NbSe_2_ via chemical synthesis, encompassing the breakdown of the precursor NbCl_5_ at heightened temperatures to subsequently engage with Se [60]. This operation involves reacting a degassed oryzamide solution with a mix of NbCl_5_ and Se under protective inert gas conditions, heated between 250 and 280 °C for a duration of 4 h. In this procedure, precursors dissolve within the solvent, resulting in a dark suspension. Post subsequent washing and heating stages, the 2D NbSe_2_ material is derived. The merits of chemical synthesis include reduced imperfections and lattice anomalies, diminished synthesis temperatures, and more affordable raw material expenditures. However, for its wide-scale adoption, chemical synthesis demands further exploration and finetuning. Often, 2D materials stemming from this method display stoichiometric discrepancies and fluctuating physical attributes.

**Figure 5:**
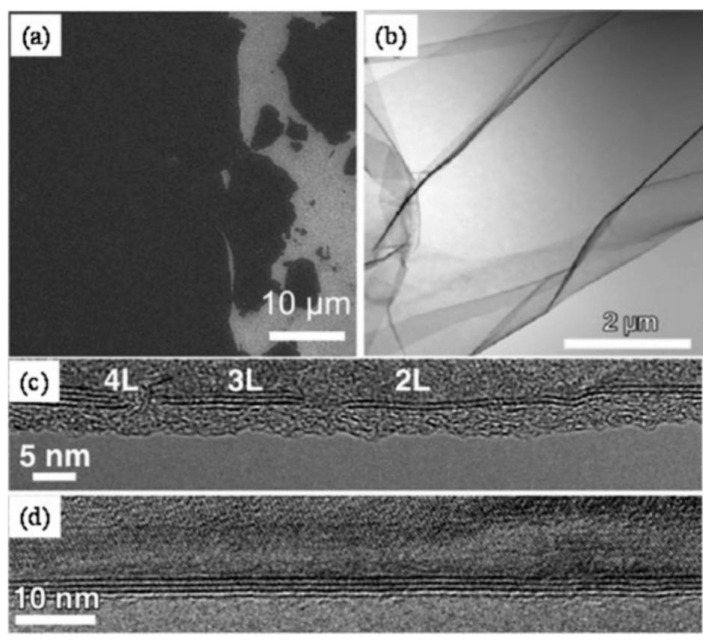
(**a**) SEM image of MoS_2_ on SiO_2_/Si substrate. (**b**) SEM image of CVD-grown MoS_2_. (**c**,**d**) Layers of CVD-grown MoS_2_ demonstrated in TEM images. Reprinted with permission from [65]. Copyright (2012) by WILEY-VCH Verlag GmbH & Co. KGaA, Weinheim.

Chemical vapor deposition (CVD) is a high-temperature chemical synthesis method used to deposit desired materials on different substrates. CVD is an extensively studied and heavily used material synthesis method that can be used to synthesize a variety of thin-film materials, including metals, semiconductors, and insulators. However, it was not until 2009, when Li et al. demonstrated the large-scale synthesis of graphene on copper foil via CVD with flow-through methane at 1000 °C, that CVD was recognized as a method that could be used to synthesize two-dimensional materials [61]. After that, several articles reported the successful synthesis of graphene on other transition metals via the CVD of hydrocarbons [62]. After this, Hwang and Fanton et al. also reported a van der Waals epitaxial growth of graphene on c-face sapphire without any transition metal catalyst [63,64]. Meanwhile, the CVD method has also been heavily used for the large-scale synthesis of uniform 2D TMDs and h-BN films. Metal sulfides are synthesized by reacting metal films and sulfur vapors at elevated temperatures under the protection of an inert gas. Zhan et al. achieved the large-scale synthesis of two-dimensional MoS_2_ via CVD [65]. MoS_2_ films were produced by exposing the Mo-coated substrate to sulfur vapor and N2 gas at a high temperature of 750 °C for 90 min, which resulted in the reaction of the Mo film with the sulfur vapor. Figure 5 shows the TEM image of 2D MoS_2_ synthesized using the CVD method [65]. The article was one of the first to report synthesizing 2D MoS_2_ on SiO_2_/Si substrates via CVD, and Zhan emphasized that the size and thickness of MoS_2_ synthesized via CVD are closely related to the substrate size as well as the thickness of the Mo film.

Metal oxide films offer an alternative avenue for creating 2D materials and can be transformed via a process known as vulcanization. This involves exposure to sulfur vapor, leading to reduction. A classic example is the derivation of MoS_2_ through the sulfur vapor reduction of MoO_3_. Herein, MoO_3_ undergoes an initial reduction when exposed to sulfur vapor at elevated temperatures, resulting in the suboxide MoO_3−x_. This then undergoes further reactions to yield 2D layers of MoS_2_. Interestingly, for MoO_3_, the necessary reduction temperature sits at a relatively lower temperature of 650 °C when contrasted with the direct synthesis of MoS_2_ from Mo. While this technique shows promise in obtaining multilayer MoS_2_ on a vast scale, generating monolayer MoS_2_ poses a challenge [66]. The impediment arises primarily because the MoS_2_ growth is stunted by an interfacial oxide layer. To address this, Lee and his team put forth a substrate pre-treatment technique ahead of MoS_2_ synthesis [66]. By pre-treating the SiO_2_/Si substrate with agents like reduced graphene oxide (rGO), tetrapotassium terephthalate (PTAS), and terephthalic acid dianhydride (PTCDA), the formation of expansive single-layer 2D MoS_2_ becomes feasible.

CVD’s crowning glory in the realm of 2D material synthesis lies in its ability to create pristine, high-quality 2D materials with tunable properties. Within the confines of CVD synthesis, the structural morphology, crystallinity, and defects of 2D materials can be fine-tuned through meticulous adjustments of the process parameters. Furthermore, the fusion of diverse precursor types (spanning gases, liquids, and solids) paves the way for novel 2D materials and heterostructures. This method also extends the possibility of doping and functionalizing 2D materials by introducing supplementary precursors during the CVD synthesis phase. However, while promising, it is pivotal to continue refining the process, especially to exercise precise control over the atomic-level properties of 2D materials and to accurately regulate stoichiometric proportions and defects.

## 3. Discussion

In the past few decades, brain-inspired computing systems based on 2D materials have been extensively studied and developed due to their unique properties and mature processing technology. These systems can be designed in various ways, with two-terminal (2T) memristors and three-terminal (3T) memristor transistors being among the most common configurations. In 2T memristors, the two-dimensional material can be used as the working/dielectric layer, and in a 3T memristor transistor, it acts as the channel/dielectric layer. This section will provide an overview of memristor devices based on 2D materials, including their materials, structures, and operating principles, and compare their memristor performance.

### 3.1. Two-Terminal Memristors Based on Two-Dimensional Materials

Strukov et al. first proposed a 2T memristor based on a two-dimensional material that can simulate the connection between presynaptic and postsynaptic neurons, where the source and drain correspond to the input and output, respectively [67]. These devices are commonly constructed in two different configurations: horizontal and vertical. In a vertical structure of a 2T memristor, the inputs and outputs are perpendicular to the channel material, while in a horizontally constructed 2T memristor, they are parallel to the channel material. The crucial component of these devices is the two-dimensional material serving as the channel/working layer. The thinness of the 2D material, consisting of only one or a few atomic layers, makes it highly suitable for constructing large-scale, low-energy array circuits. Typically, a horizontal 2T memristor consists of metal electrodes arranged horizontally on a dielectric layer and channels consisting of a two-dimensional TMD. In such a type of device, the current will flow horizontally and pass through the channel made of the 2D TMD material, causing defect migration within the channel or phase change to cause a memristive effect.

**Figure 6:**
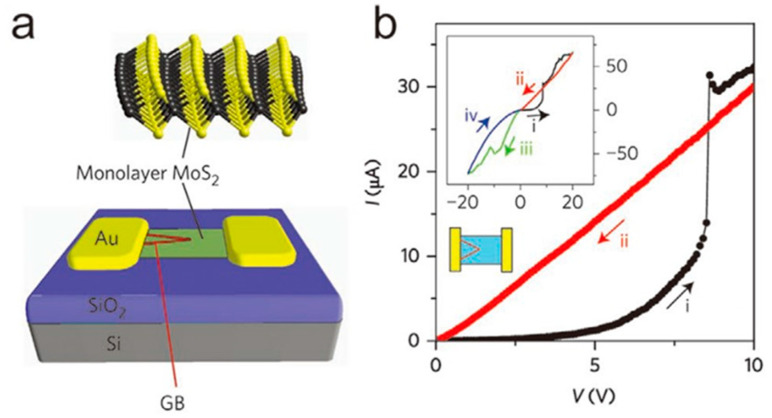

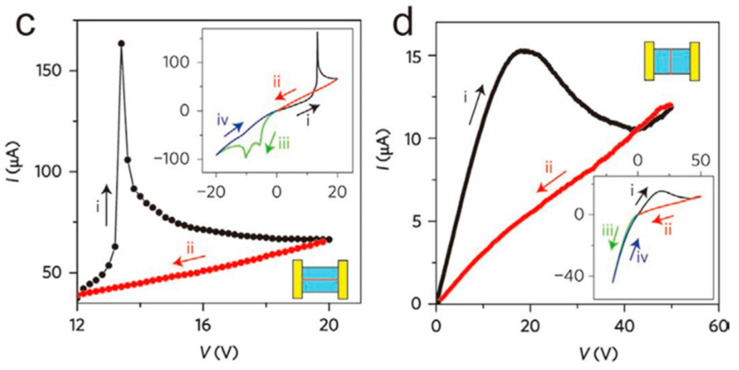
(**a**) Schematic diagram of a memristive transistor fabricated from a single layer of MoS_2_. (**b**) i–v characteristic curve of a cross-type MoS_2_ memristor. (**c**) i–v characteristic curve of a bridge-type MoS_2_ memristor. (**d**) i–v characteristic curve of an equal-partition-type MoS_2_ memristor. Reprinted with permission from [68]. Copyright (2015) by Springer Nature.

In 2018, Hersam and colleagues proposed a horizontal 2T memristor based on 2D MoS_2_ [68]. In its working state, defects within the MoS_2_ migrate to the grain boundaries (GBs), forming a low-resistance channel and thus causing a change in the resistive state of the device. As shown in Figure 6a, the device is constructed with a single layer of MoS_2_ as the channel on a SiO_2_/Si substrate, using gold (Au) as the electrodes. Based on the arrangement of the GBs within the MoS_2_ and between the two electrodes, these devices are further categorized into three types: cross types, bridge types, and equal-partition types. Each type exhibits different switching behaviors. In the first, the cross type, as shown in Figure 6b, two GBs connect to the same Au electrode and intersect within the MoS_2_ channel. The second type, the bridge type, as shown in Figure 6c, features a GB parallel to the length direction of the channel, connecting both the source and drain electrodes. The final type is the equal-partition type, shown in Figure 6d, where a GB splits the channel into two segments without connecting to any electrode. The memristive phenomena in these devices are mainly due to defect migration. During the SET and RESET processes, sulfur vacancies accumulate near the GBs, causing changes in the electrical conductivity of the MoS_2_, which results in a memristive effect. Among the aforementioned three types of devices, a cross-type memristor shows the highest resistance ratio (HRS/LRS) of 10^3^ at zero bias, and its SET voltage does not exceed 10 V.

**Figure 7:**
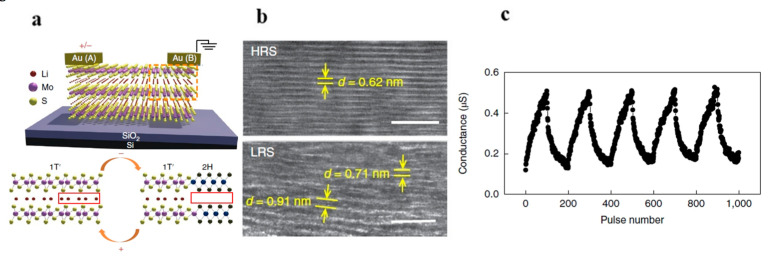
(**a**) Schematic of MoS_2_ memristor based on Li-ion insertion. (**b**) Cross-sectional HRTEM images of MoS_2_ in HRS (high-resistance state, top) and LRS (low-resistance state, bottom). (**c**) Enhancement and suppression curves formed by applying continuous pulses. Reprinted with permission from [69]. Copyright (2018) by Springer Nature.

In addition to the memristive phenomena induced by defects, phase transitions in two-dimensional materials can also lead to memristive effects. Lu and colleagues first reported an electric-field-controlled 2T memristor based on phase-change MoS_2_ in 2018 [69]. Figure 7a shows the cross-sectional view of this device, which is constructed from mechanically exfoliated MoS_2_ serving as the channel, along with gold (Au) electrodes. During operation, the device is immersed in a n-butyl lithium solution. The migration of Li ions within the channel leads to a phase transition in the MoS_2_ layer, from the 2H phase to the 1T’ phase, which results in a dramatic increase in electrical conductivity compared with the original MoS_2_. This phase transition was confirmed using Raman spectroscopy and X-ray photoelectron spectroscopy (XPS). Moreover, Raman spectroscopy verified that this phase transition is reversible. Via the movement of Li ions, the 2H and 1T’ phases of MoS_2_ can be switched, a phenomenon also confirmed using high-resolution transmission electron microscopy (HRTEM). As shown in Figure 7b, the MoS_2_ under the Au electrode expands due to the permeation of Li ions in the low-resistance state (LRS), while it contracts in the high-resistance state (HRS). This reversible ion migration leads to a change in the electrical conductivity of the MoS_2_ with the applied bias voltage in the device, as depicted in Figure 7c.

**Figure 8:**
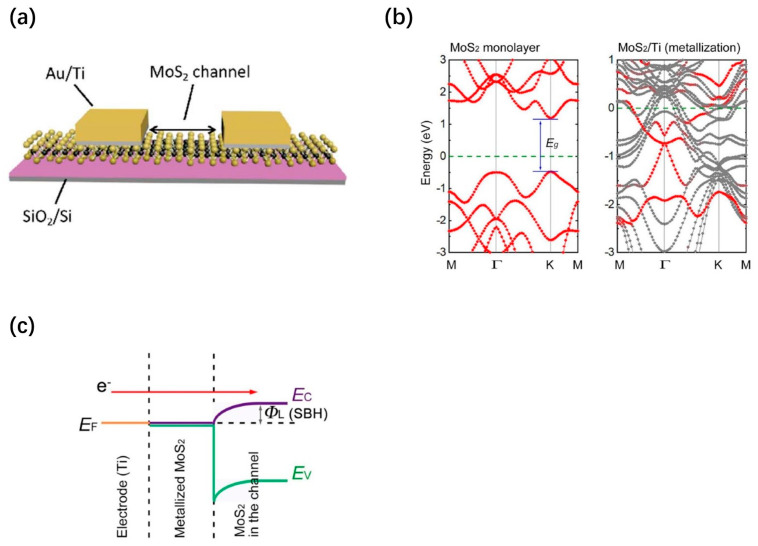
(**a**) Single-layer MoS_2_ memristor, (**b**) diagram of MoS_2_/Ti contact interface, and (**c**) band diagram of MoS_2_/m-MoS_2_/Ti structure. Reprinted with permission from [70]. Copyright (2021) by Springer Nature.

Jiaqiang Shen et al. proposed a horizontal 2T memristor based on 2D MoS_2_ in 2020, which achieved resistance tuning by aggregating field-induced sulfur vacancies at the MoS_2_/metal interface during operation. The aggregated vacancies alter the conductance of the MoS_2_ channel [70]. As shown in Figure 8a, a single layer of MoS_2_ is used on a SiO_2_/Si substrate as a channel and gold (Au) as an electrode to form the device. The device controls the sulfur vacancy migration by changing the Schottky barrier, which is different from the conductance modulation enacted with the wire filament. The MoS_2_ in contact with the Ti is metalized due to strong interactions, while the MoS_2_ in the channel retains its inherent properties. Figure 8b shows the difference in band structure between MoS_2_ and metalized MoS_2_ (m-MoS_2_). The band plot of the MoS_2_/m-MoS_2_/Ti structure indicates that there is a Schottky barrier between the m-MoS_2_ and MoS_2_ (Figure 8c). Additionally, the band plot of the MoS_2_/m-MoS_2_/Ti structure indicates bipolar resistance switching behavior, and the MoS_2_ memristor exhibits significant characteristics, such as short-term plasticity (STP), long-term plasticity (LTP), spike-amplitude-dependent plasticity (SADP), spike-time-dependent plasticity (STDP), and spike-rate-dependent plasticity (SRDP). Furthermore, the energy consumption is calculated by multiplying the results of the integration of the pulse voltage and current curve over time, where 10 pulses increase the stable post-synaptic current (PSC) and make it last for at least 5 s. The energy consumption is only 1.8 pJ. Due to the relatively high resistance during resistor holding, resulting in low read power consumption, the high resistance of single-layer MoS_2_ plays an important role in low consumption. Research has shown that monocrystalline monolayer MoS_2_ has a higher resistance than multilayer and polycrystalline silicon MoS_2_.

Vertical-structure devices are increasingly favored in modern semiconductor industries for their advantages in high-density integration [71]. These vertical-structured two-terminal (2T) memristors are usually formed of metal-oxide–metal layers. To reduce power consumption, efforts are often made to decrease the thickness of the intermediate oxide layer. However, various crystal structure problems commonly arise, leading to significant leakage currents. To address this issue, extensive research has been carried out on 2D materials. Vertical 2T memristors based on 2D materials usually have a metal/2D material/metal structure, where the current flows in a direction perpendicular to the 2D material plane. These 2D materials demonstrate controllable phase changes, defect migrations, and conductive channel formations. This combination of structural and performance advantages makes it possible to reduce the size of individual devices based on 2D materials, thereby having the potential for high-integration neuromorphic computing chips.

**Figure 9:**
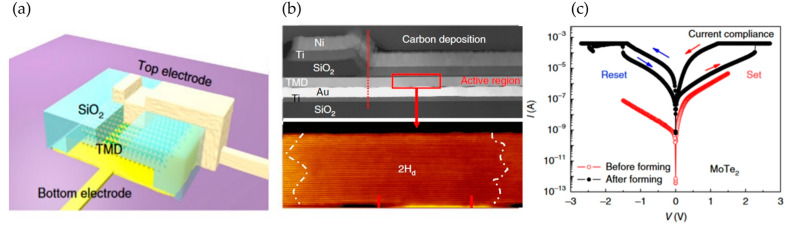
(**a**) Schematic of vertical-structure 2T memristors based on MoTe_2_ and Mo_1−x_W_x_Te_2_. (**b**) Cross-sectional STEM image showing localized phase changes. (**c**) I-V characteristic curve of this device. Reprinted with permission from [70]. Copyright (2018) by Springer Nature.

Appenzeller et al. proposed a vertical-structured 2T memristor using MoTe_2_ and Mo_1−x_W_x_Te_2_ as the memristive layers, as shown in Figure 9a–c [72]. Unlike most reported phase-change memristors, the memristive effect in this device arises due to a material phase transition from the conventional hexagonal phase (2H) to a distorted hexagonal phase (2H_d_). Conductive-AFM (C-AFM) measurements showed that the 2H_d_ phase exhibits better conductive properties compared with the 2H phase, which leads to a change in the overall resistance of the device. To study this unique phase transition, high-angle STEM was employed during the SET process. As shown in Figure 9b, the crystal structure in the middle part visibly differs from the 2H phase, specifically transitioning to the 2H_d_ phase. Meanwhile, Figure 9c shows memristive phenomena induced by phase transitions within a ± 3 V range. This research suggests that electric-field-driven reversible phase changes in transition metal dichalcogenides (TMDs) are feasible, further indicating the potential application of vertical 2T memristors based on TMDs in ultra-low power consumption fields.

**Figure 10:**
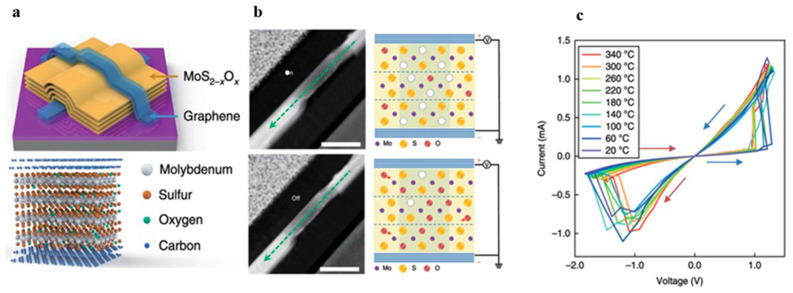
(**a**) Schematic diagram of a memristor based on a graphene/MoS_2_/graphene structure. (**b**) STEM (scanning transmission electron microscopy) images of the LRS (low-resistance state) and HRS (high-resistance state), along with a schematic showing the formation of sulfur vacancies. (**c**) Switching curves of the device in an environment ranging from 20 °C to 340 °C. Reprinted with permission from [48]. Copyright (2018) by Springer Nature.

In a vertical two-terminal (2T) memristor developed by Miao and his team, they utilized a graphene/MoS_2_/graphene structure to create the memristive device [48]. This device displayed promising memristive behavior even at high temperatures (350 °C). As shown in Figure 10, the device consists of graphene used as electrodes on both ends and a 40 nm thick MoS_2−x_O_x_ layer serving as the channel. This MoS_2−x_O_x_ layer was obtained via oxidization for 1.5 h at 160 °C. As indicated in Figure 10b, sulfur vacancies form in the channel when the device is operational. The variation in the concentration of these sulfur vacancies leads to changes in the resistance, thus forming a high-resistance state (HRS) and low-resistance state (LRS). Element changes affecting channel conductivity were observed via scanning transmission electron microscopy (STEM), and the presence of graphene also prevents metal from diffusing into the channel. Compared with conventional oxide-based memristors, this device can maintain its crystalline structure without undergoing deformation even at temperatures as high as 800 °C. As a result, as shown in Figure 10c, the device is able to maintain stable and repeatable memristive phenomena at operating temperatures ranging from 20 °C to 340 °C. The research implies that graphene/MoS_2_/graphene memristors could offer significant advantages in terms of thermal stability and functionality, making them potentially useful for applications requiring high-temperature operation. Furthermore, Huai Yang et al. proposed a non-volatile vertical structure memristor based on the heterostructure of two-dimensional room-temperature ferroelectric α-In_2_Se_3_ and WSe_2_ [73]. By applying a voltage to polarize the ferroelectricity, the resistance can be modulated between a high-resistance state and a low-resistance state, preserving the resistance state without the need for additional information storage power. To fabricate this memristor, high-quality ferroelectric α-phase In_2_Se3 crystals were synthesized using chemical vapor transport (CVT) methods. Applying a DC bias from both electrodes creates a reversible built-in electric field in the α-In_2_Se_3_ nanosheet, enabling the switching of its polarization direction between the “up” and “down” directions. To study the spontaneous polarization caused by the inverse symmetry of α-In_2_Se_3_ and the destruction of the polar structure, PFM measurements were performed on α-In_2_Se_3_ crystals, revealing two out-of-plane polarization directions with a phase difference of 180° in the α-In_2_Se_3_ sheet, corresponding to the ferroelectric domains perpendicular to the upward and downward polarization vectors perpendicular to the horizontal plane, respectively. The bandgap value α-In_2_Se_3_ is approximately 0.53 eV when the polarization direction is “up”, and the bandgap value increases to 1.09 eV when the polarization direction is reversed to “down”. A band gap of approximately 0.56 eV results in a 100-fold increase in resistance compared with the “on” state. The study shows that α-In_2_Se_3_ iron can be polarized with an electric field drive to have non-volatile memory characteristics, and it has excellent characteristics, such as a conduction/turn-off ratio of more than 100 and a relatively small switching voltage, which proves that non-volatile memory based on a vdW ferroelectric heterogeneous structure can provide new opportunities and new platforms for the utilization and development of two-dimensional ferroelectric materials for information storage.

### 3.2. Three-Terminal Memristors Based on Two-Dimensional Materials

In addition to 2T memristors, 3T memristor transistors will be discussed in this chapter. Typically, 3T memristor transistors consist of three electrodes (the source, drain, and gate), a dielectric layer, and a channel layer. Compared with 2T memristors, 3T memristor transistors can implement signal transmission and learning functions, which gives them the potential to build complex brain-like chips [74,75,76]. In a three-terminal memristor transistor, the gate serves as a control electrode to change the conductivity or impedance of the channel region by applying different voltages, enabling synaptic weight regulation to simulate the learning function in neural networks. The source and drain are used to read and write data. By controlling the gate voltage and applying the appropriate voltage pulse, it becomes possible to create a controllable state of resistance in the channel. This functionality enables the device to store and retrieve information, making it a versatile tool for data processing and storage in brain-inspired computing systems.

**Figure 11:**
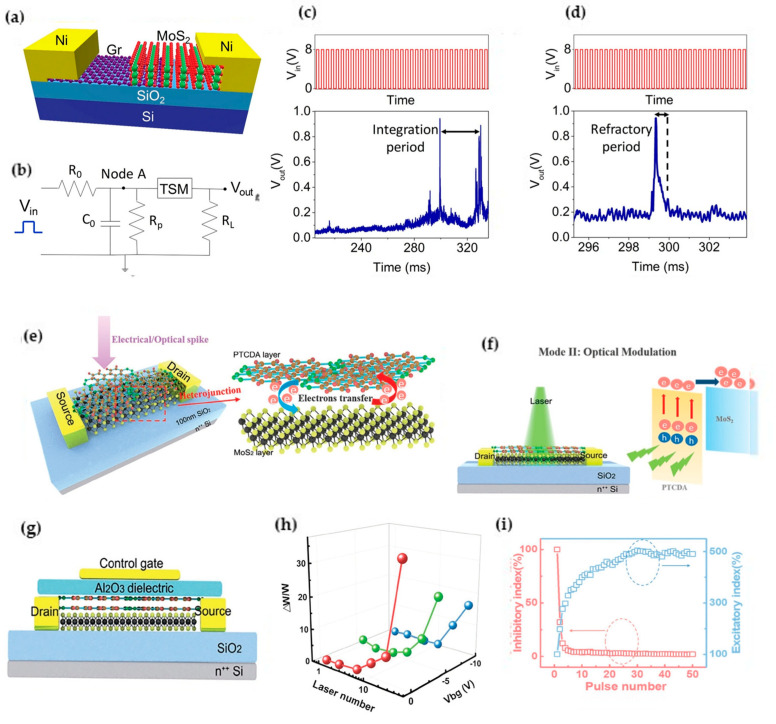
(**a**) Schematic of a v-MoS2/graphene TSM. (**b**) Schematic of the circuit used to realize the artificial neuron. (**c**) (Top) input voltage pulses of 8 V amplitude, TON = 100 µs, and frequency = 5 kHz (not to scale). (Bottom) output spike of the artificial neuron showing the integration time. (**d**) (Top) input voltage pulses of 8 V amplitude, TON = 100 µs, and frequency = 5 kHz (not to scale). (Bottom) output spike of the artificial neuron showing the refractory period. (**e**) Schematic diagram of a MoS_2_/PTCDA hybrid heterojunction modulated by an electric or optical peak. (**f**) Schematic diagram of the optical device structure and band alignment of the heterojunction. (**g**) Schematic diagram of the device architecture in electrical modulation mode neuron. (**h**) Long-term weight under different VBGs varies with laser times. (**i**) Inhibition (red)/excitability (blue) index as a function of the number of pulses. Reprinted with permission from [77]. Copyright (2019) by Springer Nature.

Hirokjyoti Kalita et al. reported a study on artificial neurons based on vertical MoS_2_/graphene threshold switch gates, as shown in Figure 11a [77]. The researchers used the volatile threshold-switching behavior of the vertical MoS_2_/graphene van der Waals heterojunction system to replicate the integral and ignition response of neurons. The threshold-switching memristor (TSM) device is connected in parallel with a resistor and a capacitor and then a resistor in series to form an artificial neuron. When an input pulse is applied to the circuit, the capacitor (Co) begins to charge. As the charge on the capacitor increases, the voltage at node A reaches the threshold voltage of the TSM, causing it to switch from an HRS to an LRS. The LRS state leads to a drop in voltage at node A. Once the voltage at node A decreases, the TSM device recovers to an HRS. This results in a decrease in the output current and voltage and a spike in the output voltage. The capacitor starts charging again, indicating the beginning of the neuron integration cycle. During the time period of neuron firing, the net charge integration in the capacitor is essentially zero, simulating the refractory period after the firing of biological neurons, and once the refractory period is over, the device returns from an LRS state to an HRS state, and the capacitor “Co” begins to integrate again. The device effectively mimics the essential characteristics of biological neurons, such as all-or-none spikes, threshold-driven action potential spikes, refractory periods after neuronal discharge, and intensity-modulated frequency responses. The random threshold-switching behavior caused by the migration of oxygen ions along the MoS2 vertical particles adds to the versatility of these artificial neurons, making them suitable for real-time computing systems based on event spikes.

Shuiyuan Wang et al. developed a high-efficiency optoelectronic dual-modulated multifunctional MoS_2_/PTCDA hybrid heterojunction synapse, as shown in Figure 11e [78]. Via the Au top gate electrode used as a control gate, carriers at the heterojunction interface are driven to simulate the separation, capture, or gradual release of neurotransmitters, resulting in inhibitory and excitatory synaptic behavior. The Au top gate electrode also serves as drain and source terminals to read the current state of the device as PSC and n + +Si back gate terminals. In electrical modulation, synaptic suppression and excitation can be achieved simultaneously in the same device through the regulation of the gate voltage, and increasing the number of gate electrical pulses can obtain a minimum suppression of 3% and a maximum boost of 500%, and the response to signals of different frequencies shows dynamic filtering characteristics. It also exhibits flexible adjustability of STP and LTP and a synaptic weight variation of up to 60, which is far superior to that found in previous work on optical modulation. All of this demonstrates the potential of all-2D MoS2/PTCDA hybrid heterojunction artificial synapses in neuromorphic computing.

**Figure 12:**
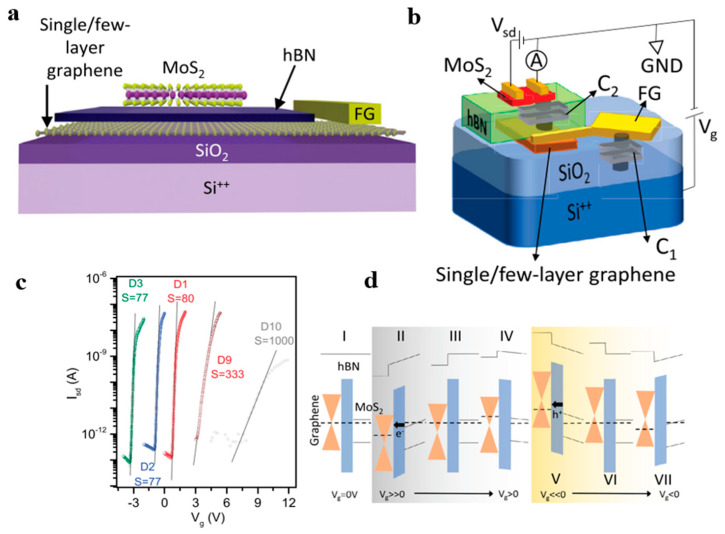
(**a**) Schematic diagram of the structure of this device. (**b**) Representative image of the gate capacitor circuit, where C1 represents the capacitance between the floating gate and Si++ (through the SiO_2_ insulation) and C2 represents the capacitance between the floating gate and the channel (hBN). FG stands for a large area of metal floating grid connected to the graphene layer. (**c**) Comparison of subthreshold slopes of devices with different floating gate configurations. D1, D2, and D3 are devices with extended floating gates, D9 without expansion of the floating gate, and D10 without floating gates. (**d**) Transport mechanism in MoS_2_ floating-gate devices. The black arrows indicate the direction of charge flow between the floating gate and the MoS_2_ channel during the enhancement and inhibition cycles. e- and h+ represent electrons and holes, respectively. Used with permission from IOP Publishing, Ltd., from [79]; permission conveyed through Copyright Clearance Center, Inc.

Paul et al. reported a MoS2/h-BN-based field-effect transistor and used graphene as a floating gate, as shown in Figure 12a,b [79]. This innovative device allows for the simulation of synaptic plasticity by leveraging the charge-tunneling effect between the transistor’s channel and the graphene floating gate. One of the significant advantages of this design is the remarkable improvement in the floating gate efficiency, which addresses the high energy consumption issue observed in conventional floating-gate transistors that require large pulse voltages. With this structure, as shown in Figure 12c, the device achieves an almost perfect subthreshold swing (77 mV/dec). In their experiments, the inhibitory and enhancement effects of synapses were successfully simulated by controlling for the carrier-tunneling effect in the h-BN insulation (Figure 12d). To further enhance the symmetry and linearity of synaptic weight updates, they also demonstrated the application of flash memory based on dual-floating gate MoS_2_/h-BN cell stacking. Via the tunneling of electrons and the control of the injection process, they achieved more controlled electron transport, which improves synaptic inhibition and enhances the symmetry of the process. The work of Paul et al. has important implications in the development of low-power, high-performance brain-like computing chips [79].

**Figure 13:**
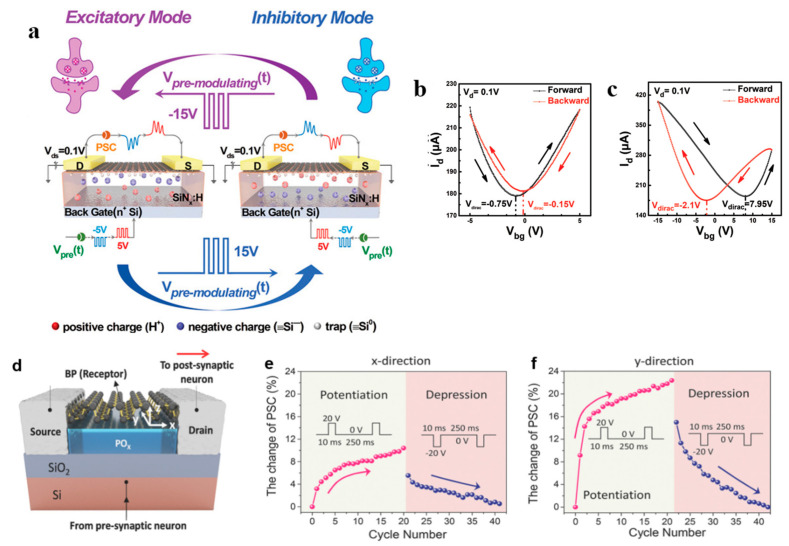
(**a**) Schematic of 3T dynamic artificial synapse based on graphene, and (**b**,**c**) are the hysteresis characteristics of this device. (**d**) Schematic of the 3T memristive transistor based on BP (**e**,**f**) shows the anisotropic carrier mobility within BP. Reprinted with permission from [80]. Copyright (2019) by WILEY-VCH Verlag GmbH & Co. KGaA, Weinheim.

Yao and colleagues reported research concerning dynamic artificial synapses based on graphene channels [80]. As shown in Figure 13a, they utilized SiN_x_:H thin films as the gate dielectric layer for graphene, achieving dynamic reconfigurations between different synaptic response modes. They introduced traps and mobile hydrogen ions in the dielectric layer to facilitate carrier trapping and the capacitive gate effect. Utilizing the carrier-trapping effect and the bipolar nature of graphene, the researchers realized spike-time-dependent long-term depression (S/LTD) and spike-time-dependent long-term potentiation (S/LTP) phenomena in the device. The hysteresis characteristics of this device can be clearly seen in Figure 13b,c. This phenomenon is attributed to the carrier-trapping effect, suggesting the existence of traps in the gate dielectric layer for capturing electrons and holes. Additionally, Yao and colleagues demonstrated that the capacitive gate effect can reconfigure the device to switch between the S/LTD and S/LTP response modes. By applying a higher voltage, the synaptic behavior can be adjusted, allowing for different response modes to the same low-intensity pulses. This research by Yao and his team showcases the potential of artificial synapses to simulate complex biological behavior, suggesting promising avenues for implementing bio-inspired computing systems.

Tian and colleagues also reported on a 3T (three-terminal) memristive transistor that utilizes the charge-trapping effect [81]. As shown in Figure 13d, this device comprises a BP (black phosphorus) layer, a PO_x_ layer, a source electrode, a drain electrode, and a back gate electrode. During the fabrication process, a 2 nm thick PO_x_ layer was first formed at the bottom of the BP layer to serve as the charge-trapping layer. The electronic behavior of the device can be controlled by applying positive and negative voltages to the silicon substrate back gate. When a positive voltage is applied to the back gate, electrons in the BP channel are captured by oxygen vacancies in the PO_x_ layer, leading to an increase in the hole concentration and current. Conversely, when a negative voltage is applied to the back gate, electrons are released back into the BP channel, resulting in a decrease in the hole concentration and current. This process of electron trapping and release simulates synaptic long-term potentiation (LTP) and long-term depression (LTD). However, unlike traditional 3T memristive transistors, this device exhibits different degrees of LTP and LTD in the x- and y-axis directions. This is due to the anisotropic carrier mobility within BP, as shown in Figure 13e,f. This suggests that the device has potential applications in directional sensing or recognition.

**Figure 14:**
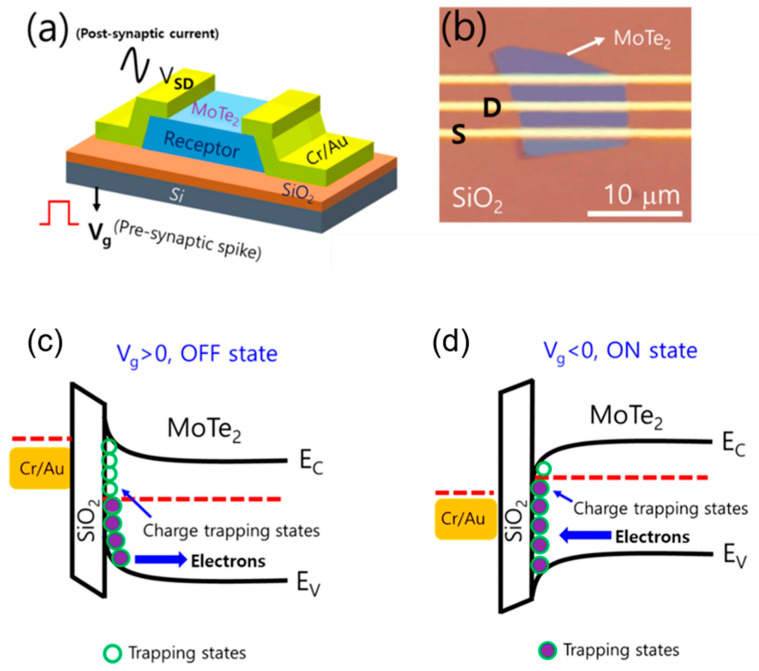
(**a**) The schematic illustration of MoTe_2_ three-terminal field-effect transistors (FETs) with charge-trapping and de-trapping dynamics at atomically thin channel 2D material/SiO_2_ interface. (**b**) The final optical microscope image of MoTe_2_ FET with Cr/Au electrodes. (**c**,**d**) Schematic band diagram of MoTe_2_ FET with trapped states at the MoTe_2_/SiO_2_ interface when negative gate is applied and when positive gate is applied.

Shania Rehman and colleagues reported on a back gate, three-terminal field-effect transistor (FET) that was prepared using MoTe_2_ as the channel material, as shown in Figure 14a,b [82]. It is worth noting that the bottom side of the MoTe_2_ flake was treated with deep ultraviolet light in ambient conditions before being transferred to the silicon substrate to promote the hysteresis loops. The study found that when the applied back gate voltage is negative (V_g_ < 0, on state; Figure 14c), the induced electric field in the SiO_2_ (dielectric) encourages the trapped electrons to be released from their trapped states into the n-semiconductor MoTe_2_ channel, producing a large current density. As a result, the conductivity of the MoTe_2_ is enhanced. On the other hand, when a positive back gate voltage pulse is applied (V_g_ > 0, off state; Figure 14d), the induced electric field depletes the electrons from the MoTe_2_, thereby reducing the channel conductivity of the MoTe_2_. By changing the conductivity of the channel by controlling the trapping and de-trapping of the charge carriers in the MoTe_2_, the device exhibits plasticity similar to that of neuronal synapses.

**Figure 15:**
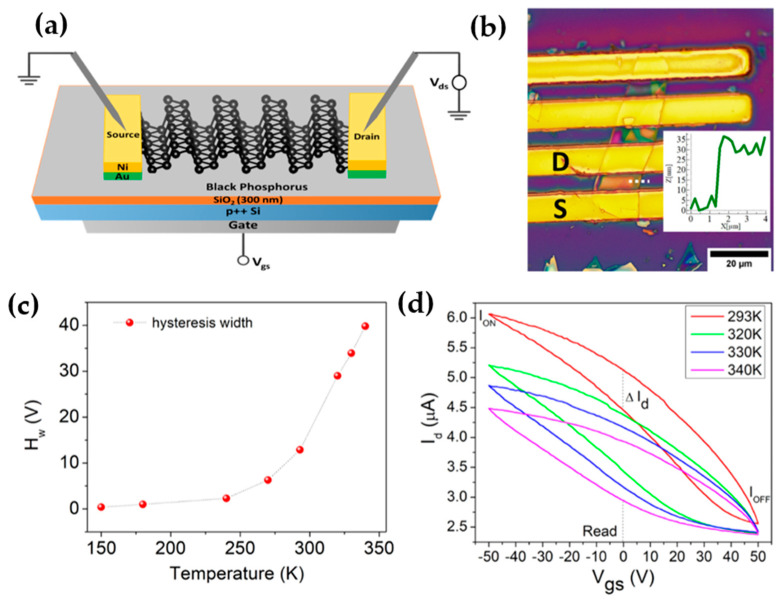
(**a**) Schematic diagram, and (**b**) an optical image of the fabricated BP transistor (the inset represents the height profile along the dotted white line obtained using AFM). (**c**) Temperature-dependent transfer width and (**d**) transfer characteristics. Original content from this work may be used under the terms of the Creative Commons Attribution 4.0 license.

In 2022, Arun Kumar and his colleagues employed a mechanical lift-off method to transfer peeled multi-layer BP thin films onto SiO_2_/p++Si substrates, thus fabricating back gate BP FET devices, as shown in Figure 15a,b [83]. Their study revealed that an increase in temperature from 150 K to 340 K resulted in a two-orders-of-magnitude increase in the hysteresis width of the device (Figure 15c). This phenomenon suggests that higher temperatures may facilitate charge trapping at the BP/SiO_2_ interface. Various defects are formed at the BP/SiO_2_ interface due to adsorbates or process residues at this junction, along with charged ions in the SiO_2_ layer. These defects create deep intragap states that act as trap centers. Trapping and de-trapping predominantly occur via tunneling, although factors such as temperature can influence the trapped charge density. When the gate pulse is in a high positive or negative state, the drain current experiences rapid increases or decreases, as shown in Figure 15d. During this process, intragap trap states are either filled with or emptied of electrons, resulting in a memory effect.

**Figure 16:**
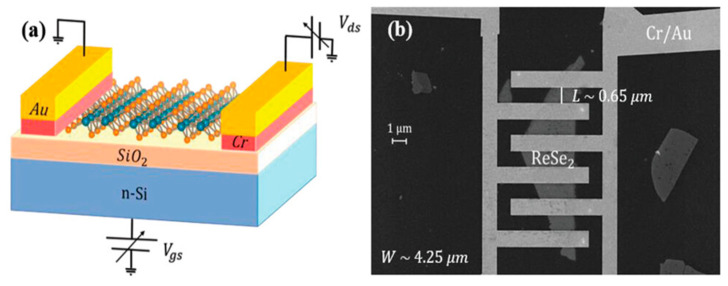
(**a**) Schematic of the device structure and the measurements set up: the ReSe2 flake is deposited onto a SiO_2_/Si substrate acting as a global back gate, and the metal contacts are Cr/Au bilayers. (**b**) Scanning electron microscope (SEM) image of the ReSe2 back-gated FET with interdigitated source/drain leads. Reprinted with permission from [84]. Copyright (2023) by the authors. *Advanced Electronic Materials* published by Wiley-VCH GmbH.

Kimberly Intonti et al. developed a pressure-enhanced, few-layer ReSe_2_ field-effect transistor, as illustrated in Figure 16a,b [84]. It exhibits a negative photocurrent (NPC) under higher vacuum conditions and a positive photocurrent in laser environments. The transition from a PPC to an NPC can be explained by considering that the light response is influenced by molecular desorption. When a single layer of ReSe_2_ is exposed to air, molecules such as H_2_O and O_2_ adsorb onto the channel surface, reducing device conductivity and mobility. Therefore, under high vacuum conditions, due to the reduced adsorption density, device conductivity and mobility increase. Although H_2_O and O_2_ molecules readily adsorb to surfaces, they are only physically adsorbed. The energy delivered by the laser is sufficient to remove them from the surface. Consequently, light has a similar effect to reducing pressure, promoting the desorption of air molecules and thereby increasing electron density and conductivity. Consequently, when the laser is activated under ambient pressure, the primary mechanism is the light-assisted desorption of electronegative molecules, leading to a return to a PPC state. At moderate pressure, both light and pressure influence the photocurrent. Since light and pressure are competing mechanisms with opposite polarities, their combined effect results in lower photocurrents. Experiments demonstrate that both air pressure and laser illumination have a significant impact on device performance. The transient response when exposed to laser pulses shows faster response times and higher light detection efficiency.

Furthermore, Das et al. presented an integrated Internet of Things platform using programmable MoS2 memtransistors capable of sensing, storing, and securing information [85,86]. The platform utilizes a single material and similar device structures, minimizing hardware costs. The energy consumption of each crypto engine is low, in the range of a few hundred picojoules, thanks to subthreshold memtransistor operation. Encrypted information remains secure even against advanced machine learning adversaries, requiring population voting for decryption. This platform demonstrates the potential for near-sensor security via in-memory bio-inspired computing.

All these applications illustrate that 2D materials hold tremendous promise in the field of neuromorphic computing. Their unique properties, such as flexibility, scalability, and tunability, make them ideal candidates for mimicking the functionalities of biological neurons and synapses. These materials enable the development of energy-efficient and high-performance neuromorphic devices that can process information in ways inspired by the human brain.

## 4. Conclusions

This review paper discussed the intrinsic characteristics of two-dimensional materials and their heterostructures, which offer unique advantages in neuromorphic computing applications. Recent reports have shown that various mechanisms inherent to two-dimensional materials can achieve the functionalities required for neuromorphic computing. Additionally, this article provided a detailed overview of the growth and preparation methods for various two-dimensional materials and the operating principles of neuromorphic devices based on these materials. The application of two-dimensional materials in 2T memristors and 3T memristive transistors showcases their potential in neuromorphic computing. Two-terminal memristors utilize two-dimensional materials as the active/dielectric layer or channel/dielectric layer, establishing connections between pre-synaptic and post-synaptic neurons in different structural designs. Vertical 2T memristors benefit from high-density integration, while horizontal ones are suitable for building large-scale low-power array circuits. The defect migration and phase transition phenomena of two-dimensional materials provide a foundation for memristive effects. Meanwhile, vertical 3T memristive transistors based on two-dimensional materials possess signal transmission and learning functionalities, paving the way for the development of complex neuromorphic chips.

Although two-dimensional materials have significant advantages compared with traditional materials, there are still many challenges in their large-scale application in neuromorphic computing. While there have been numerous reports on the successful synthesis of large-area high-quality graphene, there is still no mature method for the large-scale, high-quality growth of two-dimensional compound materials. Precise control over the microstructures of two-dimensional compounds (like TMDs), such as thickness, defects, and doping, remains a considerable challenge. These parameters critically influence the quality and performance of neuromorphic devices based on these materials. Additionally, current technologies face multiple issues in transferring two-dimensional materials while ensuring their quality. The most crucial concern is ensuring atomic-level precision and cleanliness between interfaces during the preparation of two-dimensional material heterostructures, avoiding structural and chemical inhomogeneities between the materials.

Therefore, it is essential to encourage interdisciplinary research and maintain close collaboration between scientists from various fields. Further research and development are needed on two-dimensional materials suitable for neuromorphic computing and their growth methods to enhance the performance and reliability of neuromorphic devices. Leveraging the parallel processing and energy efficiency advantages of neuromorphic computing systems will drive the development of innovative applications, enhancing computational capabilities and efficiency. There is a need to explore the application of neuromorphic devices based on two-dimensional materials in fields like artificial intelligence, simulated neural networks, the Internet of Things, and the metaverse.
